# Maternal Deaths from COVID-19 in Brazil: Increase during the Second Wave of the Pandemic

**DOI:** 10.1055/s-0042-1748975

**Published:** 2022-06-01

**Authors:** Carlos André Scheler, Michelle Garcia Discacciati, Diama Bhadra Vale, Giuliane Jesus Lajos, Fernanda Garanhani Surita, Julio Cesar Teixeira

**Affiliations:** 1 Department of Obstetrics and Gynecology, Faculty of Medical Sciences, Universidade Estadual de Campinas, Campinas, SP, Brazil

**Keywords:** COVID-19, SARS-CoV-2, coronavirus, pregnancy, acute respiratory distress syndrome, maternal death, maternal mortality, mortality rate, case-fatality, COVID-19, SARS-CoV-2, coronavírus, gestação, síndrome respiratória aguda grave, morte materna, mortalidade materna, taxa de mortalidade

## Abstract

**Objective** To compare death rates by COVID-19 between pregnant or postpartum and nonpregnant women during the first and second waves of the Brazilian pandemic.

**Methods** In the present population-based evaluation data from the Sistema de Informação da Vigilância Epidemiológica da Gripe (SIVEP-Gripe, in the Portuguese acronym), we included women with c (ARDS) by COVID-19: 47,768 in 2020 (4,853 obstetric versus 42,915 nonobstetric) and 66,689 in 2021 (5,208 obstetric versus 61,481 nonobstetric) and estimated the frequency of in-hospital death.

**Results** We identified 377 maternal deaths in 2020 (first wave) and 804 in 2021 (second wave). The death rate increased 2.0-fold for the obstetric (7.7 to 15.4%) and 1.6-fold for the nonobstetric groups (13.9 to 22.9%) from 2020 to 2021 (odds ratio [OR]: 0.52; 95% confidence interval [CI]: 0.47–0.58 in 2020 and OR: 0.61; 95%CI: 0.56–0.66 in 2021; *p* < 0.05). In women with comorbidities, the death rate increased 1.7-fold (13.3 to 23.3%) and 1.4-fold (22.8 to 31.4%) in the obstetric and nonobstetric groups, respectively (OR: 0.52; 95%CI: 0.44–0.61 in 2020 to OR: 0.66; 95%CI: 0.59–0.73 in 2021; *p* < 0.05). In women without comorbidities, the mortality rate was higher for nonobstetric (2.4 times; 6.6 to 15.7%) than for obstetric women (1.8 times; 5.5 to 10.1%; OR: 0.81; 95%CI: 0.69–0.95 in 2020 and OR: 0.60; 95%CI: 0.58–0.68 in 2021; *p* < 0.05).

**Conclusion** There was an increase in maternal deaths from COVID-19 in 2021 compared with 2020, especially in patients with comorbidities. Death rates were even higher in nonpregnant women, with or without comorbidities.

## Resumo

**Objetivo** Comparar as taxas de mortalidade por COVID-19 entre gestantes ou puérperas e não gestantes durante a primeira e segunda ondas da pandemia brasileira.

**Métodos** Na presente avaliação dos dados do Sistema de Informação da Vigilância Epidemiológica da Gripe (SIVEP-Gripe), incluímos mulheres com síndrome respiratória aguda grave por COVID-19: 47.768 em 2020 (4.853 obstétricas *versus* 42.915 não obstétricas) e 66.689 em 2021 (5.208 obstétricas *versus* 61.481 não obstétricas) e estimamos a frequência de óbito intra-hospitalar.

**Resultados** Identificamos 377 óbitos maternos em 2020 e 804 em 2021. A taxa de mortalidade por COVID-19 aumentou 2,0 vezes no grupo obstétrico (de 7,7 para 15,4%) e 1,6 vezes no grupo não obstétrico (de 13,9 para 22,9%) de 2020 a 2021 (odds ratio [OR]: 0,52; intervalo de confiança [IC] 95%: 0,47–0,58 em 2020 e OR: 0,61; IC95%: 0,56–0,66 em 2021; *p* < 0,05). Em mulheres com comorbidades, a taxa de óbitos aumentou 1,7 vezes (de 13,3 para 23,3%) e 1,4 vezes (de 22,8 para 31,4%) para os grupos obstétricos e não obstétricos, respectivamente (OR: 0,52; IC95%: 0,44–0,61 em 2020 para OR: 0,66; IC95%: 0,59–0,73 em 2021; *p* < 0,05). Em mulheres sem comorbidades, a taxa de mortalidade foi maior para as não obstétricas (2,4 vezes; de 6,6% para 15,7%) do que para mulheres obstétricas (1,8 vezes; de 5,5 para 10,1%; OR: 0,81; IC95%: 0,69–0,95 em 2020 e OR: 0,60; IC95%: 0,58–0,68 em 2021; *p* < 0,05).

**Conclusão** Houve aumento das mortes maternas por COVID-19 em 2021 em relação a 2020, principalmente naquelas com comorbidades. As taxas de mortalidade foram ainda maiores em mulheres não grávidas, com ou sem comorbidades.

## Introduction

Since the first case of COVID-19 was notified in Brazil on February 26, 2020, the pandemic caused by the Severe Acute Respiratory Syndrome Coronavirus 2 (SARS-CoV-2) has spread out at an accelerated pace. According to the Brazilian Ministry of Health, from the beginning of the pandemic up to December 09, 2021, a total of 21,184,824 people were infected by SARS-CoV-2, and 616,691 died in Brazil. 1

In the face of a viral pandemic of unpredictable and unknown evolution, the primary concern among obstetricians was whether pregnancy could be a risk factor for severe COVID-19 outcomes, as was seen for respiratory disease caused by the influenza virus in the past decades. 2 3 The first Brazilian studies using 2020 data from the Acute Respiratory Distress Syndrome (ARDS) Surveillance System (SIVEP-GRIPE, in the Portuguese acronym) reported many maternal deaths. However, they did not define whether pregnant women have a higher risk of severe outcomes than the general female population. Furthermore, early data on maternal mortality from COVID-19 were scarce and limited. 4 5 6 7

In 2020, the peak of the Brazilian pandemic occurred in the 30 ^th^ epidemiological week, when 319,653 new cases of SARS-CoV-2 infections were reported in one week. After a short period of slowdown of the pandemic in late 2020, Brazil experienced a rapid acceleration in 2021, reaching 539,903 new cases in the 12 ^th^ epidemiological week of 2021. 1 Although official obstetric data from the first and second waves have not been fully consolidated in Brazil, we have seen an increase in COVID-19 serious outcomes in pregnant women at our healthcare center, a referral hospital for high-risk pregnancy care that covers a population of > 6 million people in the Southeast of Brazil (unpublished data).

In the present study, we aim to compare death rates due to ARDS by COVID-19 in pregnant or postpartum and nonpregnant women during the first and second waves of the Brazilian pandemic.

## Methods

We conducted an exploratory analysis of death rates due to ARDS by COVID-19 in women aged between15 and 49 years old with SARS-CoV-2 confirmed by real-time polymerase chain reaction (RT-PCR) or serological antibodies. We compared pregnant and postpartum (up to 45 days after delivery) women, namely the obstetric group, with nonpregnant and nonpostpartum women, who composed the nonobstetric group. We collected data from the SIVEP-GRIPE 8 from the 8 ^th^ to the 53 ^rd^ epidemiological weeks of 2020 (February 26, 2020, to January 2, 2021) 9 and from the 1 ^st^ to the 26th epidemiological weeks of 2021 (January 3 to June 30, 2021). 10

Outcomes were defined as crude rates of death or cure among persons included in the SIVEP-GRIPE Brazilian databank. Women were also categorized according to the presence or absence of the following comorbidities: chronic respiratory disease, cardiovascular disease, diabetes, pregestational obesity and/or other conditions (immune deficiency, hematologic disease, hepatopathy, genetic syndrome, kidney chronic pathology, neurology disorder).

We also determined a maternal mortality ratio (MMR) for each Brazilian state as a proportion between the number of deaths due to ARDS by COVID-19 in the obstetric group in the years of 2020 or 2021 and the number of liveborn infants in the year of 2019, obtained from the Brazilian Information System on Live Births (SINASC, in the Portuguese acronym). 11 Only births from women aged between 15 and 49 years old were considered.

The detailed database used in the present study is available at the Mendeley Data repository. 9 10 We evaluated public and unidentified data from a national database that did not require prior ethical committee approval.

We calculated the mean age between the 2 groups and compared it with the Student t-test and the odds ratio (OR) with a 95% confidence interval (CI) to compare mortality. We used the software EPI-Info version 7.2.2.16 (Centers for Disease Control and Prevention, Atlanta, GA, USA).

## Results

We identified 47,768 women with ARDS by COVID-19 in 2020, of which 4,853 were from the obstetric group and 42,915 were nonobstetric. In the first 6 months of 2021, we identified a total of 66,689 female patients, of which 5,208 were from the obstetric group and 61,481 were from the nonobstetric group ( Table 1 ).

**Table 1:** Association between acute respiratory distress syndrome by COVID-19 and final outcomes in the obstetric and nonobstetric groups, with or without comorbidities

Group	ARDS/COVID-19 in 2020 (8 ^th^ to 53 ^rd^ weeks)				ARDS/COVID-19 in 2021 (1 ^st^ to 26 ^th^ weeks)				Death rate 2020–2021
	Deaths (%)	Cure (%)	OR (95%CI)	*n*	Deaths (%)	Cure (%)	OR (95% CI)	*n*	
All Women									
Obstetric	377 (7.7)	4476 (92.2)	0.52 (0.47–0.58)	4853	804 (15.4)	4404 (84.6)	0.61 (0.56–0.66)	5208	2.0
Nonobstetric	5946 (13.9)	36969 (86.1)		42915	14073 (22.9)	47408 (77.1)		61481	1.6
Women with comorbidity									
Obstetric	190 (13.3)	1239 (86.7)	0.52 (0.44–0.61)	1429	487 (23.3)	1598 (76.6)	0.66 (0.59–0.73)	2085	1.7
Nonobstetric	4376 (22.8)	14804 (77.2)		19180	8824 (31.4)	19280 (68.6)		28104	1.4
Women without comorbidity									
Obstetric	187 (5.5%)	3237 (94.5%)	0.81&&(0.69–0.95)	3424	317 (10.1%)	2806 (89.9%)	0.60&&(0.53–0.68)	3123	1.8
NonoObstetric	1570 (6.6)	22165 (93.4)		23735	5249 (15.7)	28128 (84.3)		33377	2.4

Abbreviations: ARDS, acute respiratory distress syndrome; CI, confidence interval; OR, odds ratio.

In the 10 pandemic months of 2020, we identified 377 (7.7%) deaths of pregnant or postpartum women (OR: 0.52; 95%CI: 0.47–0.58), whereas, in the first 6 months of 2021, this number increased to 804 (15.4%; OR: 0.61; 95%CI: 0.56–0.66), as shown in Table 1 . Overall, we observed an increase in women deaths from 2020 to 2021: from 7.7 to 15.4% (2.0-fold) in the obstetric group, and from 13.9 to 22.9% (1.6-fold) in the nonobstetric group ( Table 1 ).

Considering the concurrent medical conditions, we observed an OR increase from 0.52 (95%CI: 0.44–0.61) in 2020 to 0.66 (95%CI: 0.59–0.66) in 2021, which not happened with the group of women with no comorbidities. We observed that the increase in deaths was more pronounced in the obstetric group (1.7-fold; from 13.3 in 2020 to 23.3% in 2021) than in the nonobstetric group (1.4-fold; from 22.8 to 31.4%). However, the inverse was observed for women without comorbidities, in whom the increase in the number of deaths was more expressive in the nonobstetric group (2.4-fold; from 6.6% in 2020 to 15.7% in 2021) compared with the obstetric group (1.8-fold; from 5.5% in 2020 to 10.1% in 2021).

**Fig. 1:**
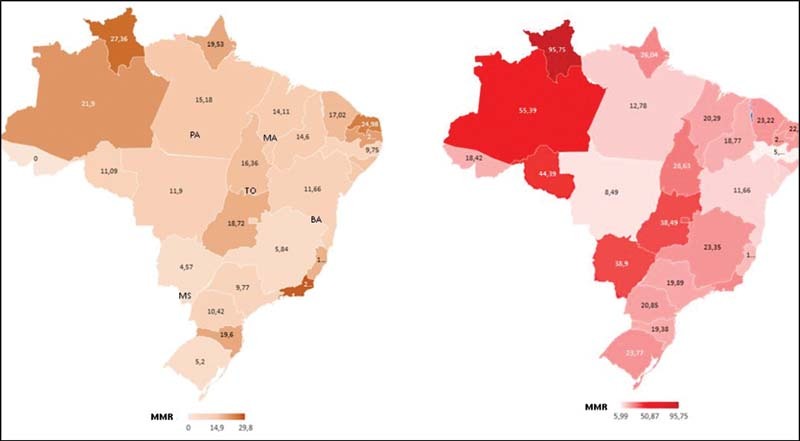
Maternal mortality ratio (MMR) by acute respiratory distress syndrome (ARDS) by COVID-19 in each Brazilian state for the years of 2020 (February to December) and 2021(January to June).

As illustrated in Figure 1 , there was an expressive increase in MMR by COVID-19 in 2021 in almost all Brazilian states. Nonetheless, this increase was most prominent in the North of Brazil, specifically in the states of Amazonas and Roraima, the epicenter of the P.1 emergence.

Moreover, nonpregnant women enrolled in the present study had a significantly higher mean age than pregnant and postpartum women ( *p* < 0.0001, t-test; data not shown in tables). This difference was observed both in the 2020 (32 versus 29 years old) and 2021 study periods (38 versus 29 years old).

## Discussion

### Main Findings

The main findings of the present study were: 1) maternal death rates more than doubled in the second pandemic wave in 2021 ( *n* = 804), even considering a shorter period that corresponded to nearly half of the one analyzed in the first wave in 2020 ( *n* = 377); 2) there was a remarkable increase in maternal mortality ratio by COVID-19 in 2021 in almost all Brazilian states, after the emergence of the P.1 variant; 3) this increase in mortality was prominent in pregnant or postpartum women with comorbidities; 4) in both periods, deaths were higher in nonpregnant women than in pregnant or postpartum women.

### Results in Context with the Scientific Literature So Far

When interpreting any evidence of the impact of COVID-19 on pregnancy, some considerations should be made. Studies focusing on the effects of COVID-19 on pregnant women are often based on symptomatic patients, thus underestimating the rates of unfavorable outcomes in women without COVID-19. Equally scant is the evidence from studies comparing pregnant with nonpregnant women with severe forms of COVID-19. 12 13 14

Our results are in agreement with data from the literature showing that COVID-19 is a sufficient factor for serious clinical outcomes in pregnant/postpartum women and for worse neonatal outcomes. 15 A prospective cross-sectional study conducted at the beginning of the pandemic with a smaller sample size showed an increase in adverse neonatal outcomes in pregnant women with COVID-19 compared with pregnant women without COVID-19, especially in pregnant women with comorbidities. 16 Although the database we used does not provide information on pregnant women without COVID-19, we show a significant rate of maternal deaths by COVID-19, especially in patients with some comorbidity.

A meta-analysis conducted by Khalil et al. 17 showed that maternal intensive care admission due COVID-19 was higher in cohorts with higher comorbidity rates. A small cohort of pregnant patients with initial asymptomatic COVID-19 showed that the development of severity appears similar to that found for nonpregnant women. 14 In our study, the mortality rates of nonpregnant women were higher than those of the obstetric groups in all conditions analyzed. Even so, the increase in maternal mortality from 2020 to 2021 that we found is an important factor, since pregnant women constitute a vulnerable group with restricted access to medications and generally do not participate in clinical research to combat COVID-19, such as vaccine trials.

Comorbidity appeared to be a more significant risk factor for COVID-19 in the nonobstetric group than in its counterpart. Moreover, nonpregnant women enrolled in the present study had a significantly higher mean age than pregnant and postpartum women. We observed this difference in the 2020 (32 versus 29 years old) and 2021 study periods (38 versus 29 years old). It is well-known that aging is associated with worse COVID-19 clinical outcomes, as was demonstrated by Khalil et al., 17 who indicated that maternal intensive care admission was higher in cohorts with maternal age > 35 years. In our study, the average age difference between the obstetric and nonobstetric groups may be a confounding factor on the influence of comorbidities in older women from the obstetric group. An extension of the present study, adjusting the effect of age and comorbidities in the analyzed groups, is currently being outlined by our research team.

Recently, the world has followed with concern the healthcare system collapsing in the city of Manaus, the capital of the state of Amazonas, due to the upsurge of the COVID-19 pandemic. The raised hypothesis of the emergence of a more transmissible variant was confirmed by Faria et al., 18 who identified a novel SARS-CoV-2 variant of concern in samples collected in November and December 2020 in Manaus. The so-called P.1 lineage acquired 17 mutations, including a trio in the spike protein (K417T, E484K, and N501Y). These researchers also estimated that the P.1 lineage may be more transmissible and more likely to evade protective immunity elicited by previous infection with non-P.1 lineages.

Although the SIVEP-GRIPE database does not provide information on the virus variant, the acceleration of the Brazilian pandemic observed in late 2020 and early 2021 coincides with the identification of the P.1 lineage. The spread of the newly mutated virus throughout the Brazilian population, consequently infecting more younger women, probably reflected in the observed increase in maternal deaths. As illustrated in Figure 1 , there was an expressive increase in MMR by COVID-19 in 2021 in almost all Brazilian states. Nonetheless, this increase was most prominent in the North of Brazil, specifically in the states of Amazonas and Roraima, the epicenter of the P.1 emergence. As a comment, in our hospital, there were no deaths in pregnant women in 2020 and, in 2021, 5 deaths in until April. These five cases were positive for SARS-CoV-2; RNA sequencing identified four of them as the P.1 variant and one was identified as a British variant (unpublished data).

### Implications for Clinicians and Public Health Managers

The national pandemic scenario presented herein highlights the need for coordinated strategies to contain the pandemic and avoid further spreading of other potentially dangerous variants. Brazil is a continental country, with ethnic, social, cultural and economic disparities that impact access to the health system in different regions. The irregular distribution of the number of deaths throughout the Brazilian states is probably related to different patterns of population exposure and to the deficient infrastructure available to assist severe cases.

Pregnant women represent a unique and vulnerable group. In this context, our study provides crucial epidemiological information for the scientific community and public health managers about the impact of the COVID-19 pandemic on pregnant women. We analyzed data from an extended period, allowing the evaluation of the Brazilian pandemic evolution from its onset in 2020 to its worsening in 2021, the latter coinciding with the emergence of the P.1 variant in Manaus.

Knowledge about the benefits of vaccination in mitigating the pandemic is a consensus all over the world. In Brazil, 63,276,223 people received all the doses prescribed by the vaccination protocol until September 7, 2021. 19 Although there is no consolidated information on the efficacy and safety of COVID-19 vaccines in pregnant women, current guidelines recommend their administration even during pregnancy, as the risks of COVID-19 outweigh the hypothetical risks of the vaccines. 20 Vaccination against SARS-Cov-2 in Brazil started on January 17, 2021, in the priority groups defined by the National Immunization Program of the Ministry of Health. 21 In May 2021, pregnant and postpartum women with comorbidities were included in this priority group, which is consistent with the concerns raised by our results on maternal mortality.

The present study is based on a comprehensive public database that provides information on large numbers of women. Epidemiological information about the deaths of pregnant women is particularly important for the scientific community and public policy managers to fight this pandemic in Brazil.

The present study shows findings that raise the need for actions to protect the health of all women during the pandemic, since COVID-19 is an important cause of women deaths in Brazil, especially in second wave after the emergence of the P.1 variant of SARS-Cov-2. The increase in maternal deaths in 2021 fosters the debate on the expansion of coverage and acceleration of the vaccination in pregnant women before more dangerous SARS-CoV-2 variants emerge.

### Strengths and Weaknesses of the Study

The present study is based on a comprehensive public database on COVID-19, which allowed us to include a large sample size comprising pregnant and postpartum women and compared them with nonpregnant women, all hospitalized with the severe form of COVID-19 confirmed by laboratory and clinical tests.

Although notification of COVID-19 is mandatory in Brazil, we cannot guarantee that the databank covers all women hospitalized due to COVID-19. Data entry is done manually by health professionals throughout the national territory; therefore, there is probably a significant amount of data gaps.

The present study is a comparative analysis of the number of deaths due to COVID-19 between obstetric and nonobstetric women in the years of 2020 and 2021, in the presence or absence of comorbidities. The inclusion of a postpartum group could bias the data analysis, as there is the possibility that some pregnant women with viable fetuses could have been hospitalized with ARDS and underwent childbirth due to the worsening of the clinical condition. Eventual deaths are registered as having occurred in the postpartum period when they resulted from the evolution of the disease since pregnancy. The public database on which the present study is based did not have the necessary data for this evaluation.

## Conclusion

There was an increase in maternal deaths from COVID-19 in 2021 compared with 2020, especially in patients with comorbidities. Death rates were even higher in nonpregnant women, with or without comorbidities.
